# Supplementary material for: The development and evaluation of a mHealth, community education and navigation intervention to improve clinical breast examination uptake in Segamat Malaysia: A randomised controlled trial
Source: PLoS One. 2023 Oct 5;18(10):e0288437. doi: 10.1371/journal.pone.0288437 (PMC10553222; doi:10.1371/journal.pone.0288437)
Supplement: S2 File — (PDF) [file pone.0288437.s003.pdf]

Title: Improving early detection of breast cancer in Malaysia during the COVID-19 pandemic - the use of mHealth to improve community education and navigation: a study protocol

Abstract

Design and objective: This randomized controlled study aims to evaluate an intervention in Malaysia that educates women about breast cancer (BC) screening and navigates them to Clinical Breast Examination (CBE) services and leads to an increase in BC screening uptake whilst taking account of the COVID-19 pandemic context.

Study population: Women who are aged 40-74 years, living in Segamat District, Malaysia, with a registered mobile phone number will be sampled randomly from the total population of women who participated in the South East Asian Community Observatory (SEACO) Health Survey (2018). BC patients or women experiencing BC symptoms at the time of recruitment will be excluded. Computer-generated random numbers will be used to assign women randomly to the intervention group (IG) or the comparator group (CG).

Interventions: The IG will receive a multi-component mHealth intervention i.e. information about BC will be provided through an educational website, and telephone calls and text messages from community health workers (CHWs) will raise BC awareness and offer to navigate women to CBE services. The usual free opportunistic screening service will be available for CG participants. It will not be possible to 'blind' participants, data collectors and CHWs.

Outcome measures: CBE screening uptake is the primary outcome measure and will be recorded by nurses conducting CBEs. Data collectors will use telephone interviews to assess differences in relation to awareness about BC signs/symptoms, intention to attend CBE and mammogram screening, breast self-examination practice and barriers to screening pre- and post-intervention. IG participants will provide an assessment of intervention acceptability and appropriateness.

Analysis: A Chi-square test will be used to compare the proportion of participants who attended CBE within 4-6 weeks after the baseline survey in the IG and CG. Multivariate regression analyses will be conducted to investigate between-group differences over time and variable influences on screening participation.

Background

Breast cancer (BC) is the most common cancer in Malaysia with an age-standardized incidence rate (ASR) of 34.1/100,000 (2012-2016) [1] and an age-standardised mortality rate of 12.0/100,000 (2020) [2]. Prior to the COVID-19 pandemic, it was estimated that 1 in 10 males and 1 in 9 females will develop cancer before 75 years old [1] and that about one-third to one-half of cancer-related deaths could be avoided through early presentation, detection and appropriate treatment. BC patients were diagnosed at advanced stages (48%) [1] with a considerable variation in BC incidence and prognosis across ethnic groups and geographical areas in Malaysia. For example, although BC incidence was lowest among ethnic Malay women [1], late presentation and poorer survival was more prevalent among this ethnic group compared to others [3]. Women from rural areas also presented at later stages, i.e. the majority (60%) of BC patients from rural areas of Segamat district presented at stages III and IV [4].

According to the World Health Organization (WHO), early detection, treatment and management of BC are the three pillars of BC control [5,6]. Health promotion and early detection are key in LMICs where late-stage detection of BC is high [6]. The most common screening modalities are clinical breast examination (CBE) and mammogram. CBE is less cost-intense than mammograms and evidence suggests that CBEs are as effective as a mammograms in terms of mortality [7]. In Malaysia, opportunistic CBE and mammography are the two main methods of BC screening [8]. Consequently, BC screening uptake depends on doctors offering CBE and mammogram when women at average risk of BC attend the clinic and/or women being aware of BC and the importance of screening and early detection.

CBE uptake in Malaysia varies between the main ethnic groups (Malay, Chinese, Indian and indigenous women) and ranges from 37% among indigenous women to 66% among Chinese Malaysians [9]. A recent survey of 993 women in Selangor State showed that only 29.7% of study participants aged ≥50 years attended BC screening [10]. A quarter of the sample did not know 'how to go about BC screening', understanding about mammograms was lacking and willingness to participate in BC screening was low [10]. Negative beliefs and attitudes towards cancer and screening, lack of time and confidence to visit a doctor were also common barriers to help-seeking [10].

Lack of awareness about cancer signs is one of the key risk factors for patient-related delay in receiving a diagnosis [11,12], and difficulty in accessing a doctor was previously associated with anticipated delayed help-seeking for breast changes [12]. These factors may help to explain why BC patients in poor rural communities in Malaysia tend to be diagnosed at advanced symptom presentation [4]. Thus, providing easy-to-understand education and addressing access barriers is key in improving cancer screening uptake. Often, community health workers (CHW) are trained to engage with communities in low- and middle-income countries (LMICs) where resources are limited and commonly play a role in educating and assisting individuals to access health services [13,14] For example, a recent review

highlighted the role that CHWs play in promoting cervical cancer screening [13] and, in the response to the COVID-19 pandemic, CHWs have delivered testing and vaccination programmes in collaboration with local authorities, particularly in LMICs with less resourced health care systems [15].

The COVID-19 pandemic and the resulting movement control orders (MCOs) in Malaysia, which enforced travel restrictions to limit the spread of the virus, add further barriers to accessing health services, particularly amongst rural communities, and contribute potentially to an increase in BC cases and their late detection. Globally, the pandemic is increasing the use of mobile health (mHealth) and telehealth for the management of the pandemic and the delivery of healthcare [16–18]. The WHO supports the adoption of a 'global strategy on digital health' [19] to contribute to the advancement of the sustainable development goals [20]. Digital health is also likely to aid in the task of navigating at-risk individuals to use cancer screening services [21,22]. Our main aim is to design, implement and evaluate an intervention to improve uptake of CBE screening in Malaysia and BC symptom recognition in the context of the COVID-19 pandemic.

Conceptual and theoretical framework

In keeping with the UK MRC Framework for the Development and Evaluation of Complex Interventions [23], we conducted a series of research activities before preparing this protocol for a RCT of the intervention. We reviewed and synthesised evidence and experiential learning regarding the implementation of community navigation and mHealth education programmes for BC early detection and diagnosis in LMICs; assessed the views and preferences of key stakeholder groups regarding community navigation, specifically the views of women in rural communities; and co-designed a culturally sensitive education and navigation programme for Malaysia. These investigative activities and results are outlined below in terms of informing the protocol for our RCT study.

The evidence synthesis that informed the intervention design was informed by a rapid review of reviews, quantitative surveying of the target population about their BC screening practices and qualitative interviews and focus group discussions (FGDs) with the target population and key stakeholders. The Behaviour Change Wheel (BCW), a theoretical framework that is used widely to guide the design of behavior change interventions, including interventions to improve early cancer diagnosis [24,25], was applied to the intervention design process [26]. We mapped the results from the evidence synthesis (e.g. facilitators and opportunities) onto the COM-B (Capability, Motivation and Opportunity – Behaviour) [27] core of the BCW (**Table 1**) to inform the development of the intervention.

Rapid review of reviews

The rapid review of reviews assessed the implementation and effectiveness of mHealth interventions to increase awareness about cancer screening and screening uptake [28]. Text messages and phone calls were the most commonly utilized mHealth methods to contact prospective participants. However, the overview indicated that text messages or email alone were ineffective in increasing screening uptake. Successful cancer screening uptake interventions combined at least two modes of communication, facilitated or offered screening appointments for participants, alone or in combination with education, patient navigation and/or addressed negative beliefs of participants (similar to findings from recent review of CRC screening uptake in LMICs [29]). The overview identified also a lack of navigation in cancer service delivery and a need to improve two-way communication between patient and service provider. Text message reminders, too, enhanced screening uptake. Conversely, research about mHealth interventions in vulnerable populations was sparse.

Qualitative research

We conducted four FGDs with 22 women from the major ethnic groups in Malaysia and interviews with 22 key stakeholders including nurses, doctors, radiologists and volunteers from the Breast Cancer Support Society Segamat. The qualitative research explored attitudes, barriers and beliefs about BC screening, challenges in breast health service delivery and how gaps in BC screening and early diagnosis in rural Malaysia might be addressed. A lack of resources (e.g. female radiographers and doctors, equipment, subsidized mammograms, and private CBE/mammogram facilities) was noted to be a challenge for primary and secondary care services. Often, screening programmes targeted women who attended mother-child clinics rather than at-risk women. Community-related challenges included lack of knowledge about BC and screening, preferential trust in traditional medicine, lack of support from husbands and fear of marital separation, apprehensions about diagnosis and treatment (breast removal), embarrassment about breast-related issues and travel distance to screening facilities. Participants recognized the importance of educating women to address the fear and embarrassment about screening and BC and to highlight the importance of early detection. Additional noted supports included subsidized screening, health talks and/or support groups for women and the involvement of non-governmental organizations.

Quantitative research

In 2017, we run a survey of 250 women aged 40 years and above, who were randomly sampled to represent women from Segamat district, which confirmed low screening awareness and uptake [30]. Only 53% and 47% of women heard of CBE and mammogram, respectively, and 36% and 22% received

the respective screening test. Half of the sample endorsed CBE (50%) and mammogram (44%) as beneficial and only 46% were aware that their nearest clinic offered CBE screening. The majority of participants lived less than 30 minutes away from clinics that facilitated CBE screening (74%) though only 26% lived near a mammogram facility. Barriers to attending BC screening were stigma, fears related to diagnosis and surgery, breast examination by a male doctor, delay in getting an appointment, cost of screening and distance to healthcare facility. There were major differences between ethnic groups, e.g. significantly more Malays reported perceived barriers compared to other ethnic groups (i.e. fear of diagnosis, lack of family support, lack of time, cost of screening, distance to health clinic and fear of pain and discomfort).

Study design and methods

This section presents the intervention and the plan for the implementation of the randomised study and outcome measures that will be used to evaluate the intervention.

Setting

Malaysia is a multi-ethnic country with a population of 32.7 million in 2020 [31]. The main ethnic groups are Malays (69.6%), Chinese (22.6%) and Indians (6.8%) [31] and the majority of the population (77%) live in urban areas and 23% live in rural areas [32]. The South East Asian Community Observatory (SEACO) is a health and demographic surveillance system (HDSS) established in 2011 by Monash University and is located in Segamat, a district in the state of Johor, Peninsular Malaysia. SEACO has a comprehensive database of over 44,000 people living in Segamat, Malaysia, which is 85% of the population in 5/11 sub-districts (Sungai Segamat, Jabi, Gemereh, Bekok, and Chaah) and 24% of the total population in Segamat. It captures detailed longitudinal information about the health of Segamat residents; and provides a research platform for a focused study of screening implementation [33]. Participants will be recruited from two sub-districts, i.e. Sungai Segamat – a town area, and Jabi – a rural area, which contain a mix of ethnic groups, and are representative of the population in Segamat. SEACO conducts three main types of surveys: 1) An annual survey to collect a range of demographic indicators 2) a full census survey, conducted every 5 years, that includes a more detailed questionnaire than the annual survey and 3) a health survey, conducted every 5 years, to examine the health profile of those aged ≥35 years regarding illness, health services use, mental health, quality of life etc.

Study population

The target population are residents from Sungai Segamat and Jabi who completed the health survey in 2018, are recorded in the SEACO database and have previously given consent to be contacted about participating in research studies. We will contact women aged 40-74 years who have registered a phone number with SEACO. Only women who will be able to provide a mobile phone number when contacted will be able to participate in the study. BC patients and survivors will be excluded from this study. Women who report to the researchers at the time of the interview that they are experiencing BC symptoms will be excluded from the research and encouraged to seek help from their local doctor as soon as possible.

Participant randomization and recruitment

Women will be selected randomly from the two sub-districts using the SEACO database as the sampling frame and then randomized to the intervention group (IG) or comparator group (CG) with a 1:1 allocation prior to being contacted. Randomisation will be conducted by a SEACO statistician (R.I.) using Stata module RANDOMIZE without blocks [34]. Participant enrolment will be conducted by trained SEACO data collectors. All randomly sampled women who were then randomized to the IG or CG will be invited to participate by trained data collectors over the phone. The resources for this study do not permit separation of enrollment and data collection procedures and, so, DCs will be aware which group the women are assigned to and this aspect may increase the risk of bias. Participants and intervention providers will not be blinded during the enrollment and surveys. CHWs will not collect outcome measures. Women who agree to participate will receive an explanatory statement about the study and a token of appreciation for their participation.

Intervention

The evidence synthesis presents a clear narrative with respect to the need to educate rural women about BC screening and the importance of early detection of cancer combined with community navigation to encourage women to attend BC screening. IG and CG participants will be interviewed as baseline and at follow-up four weeks after intervention delivery has been completed.

Women who will be randomized to the IG will receive an intervention consisting of mHealth education and community navigation. Our previous mass media campaign, the Be Cancer Alert Campaign (BCAC), improved BC symptom awareness in Malaysia [10] and the BCAC website, video and brochure will be used to educate women as part of this study. Women face numerous emotional, practical, socio-cultural and health-system related barriers, and most recently, concerns related to the COVID-19 pandemic. We will train local female community health workers (CHWs) who will offer women two-way communication to address their fears and help them navigate the breast health care

system [35]. Additionally, we will train 1-2 male CHWs to engage with a woman's husband or male relatives if a woman communicates to her female CHWs that she would benefit from such engagement. There are a number of health system-related barriers and we will use the family planning clinic ran by the Ministry of Women, Family and Community Development (LPPKN) in Segamat to refer women for free CBE screening. A one-off reimbursement for transportation fees to the clinic will be offered to participants. Due to the COVID-19 pandemic and the MCO in Malaysia, the uptake intervention will be delivered through mHealth. A multi-component mHealth intervention will be used to promote screening and will include a combination of telephone calls and text messages to communicate with, and navigate, participants.

Intervention Group

Figure 1 demonstrates the intervention flow. Trained data collectors from SEACO will recruit women assigned to the IG via phone call and conduct a baseline survey with women who provide verbal consent to participate in the study. Data collectors will then send women from the IG group a text message that contains the link to the BCAC materials [36]. One CHW will be assigned to support between 50-60 women who agreed to participate. CHWs will call the women they have been assigned to in the same week to discuss BC symptoms and BSE, address any barriers to screening, and ask them whether they are interested in attending a CBE at the LPPKN clinic. If the participants are interested, CBE appointments will be offered to take place the following week at a time that suits the participant. CHW will address concerns of participants who are not interested in screening, but no further action will be taken by the CHW if participants refuse to attend the CBE. Nurses will ask women with normal CBE results to attend screening biannually as recommended in the clinical practice guidelines [37]. Women with abnormal findings during the CBE will see a doctor at the LPPKN clinic who will refer them to a hospital for a mammogram as soon as possible after their CBE, free of charge. Participants will be informed in private about their mammogram results by the LPPKN nurses and CHWs will not be informed of the result. CHWs will call women who do not attend the CBE to identify the reason for non-participation, address further barriers and reschedule the appointment if agreed with the participant. CBE positive women who miss their mammogram appointment will be called by the LPPKN nurses.

Recruitment and training of CHW

We aim to recruit 15-20 CHWs from the two study sub-districts. We will approach women aged 40-74 years from the SEACO community engagement committee (CEC) and ask them to participate as well as to identify other women who might be willing to be trained as a CHW for this study. CHWs will be recruited from different ethnic backgrounds to be matched with up to 60 female participants with the

same ethnic background and mother tongue (Malay, Cantonese, Hokkien, Tamil and English), which has been demonstrated to be more effective in increasing mammography screening uptake [38]. A recent review of CHWs reported that CHW-led interventions were associated with and increase in CBE and mammogram screening rates in 70% of the included research papers [14]. The CHW will be asked to confirm, prior to contacting the matched participants, that they have no pre-existing formal relationship with the participants (e.g. family relationship, teacher-student relationship, etc.), however, the CHW may be friendly with participants who live in the same neighborhood. CHWs will need to be literate, have access to a mobile phone and are able to attend all training sessions. CHWs will be asked to attend five hours of virtual training delivered over five consecutive weeks. A breast cancer surgeon, a public health physician, a staff nurse trained in health communication, a LPPKN nurse and a public health academic will train the CHWs on the basics of BC, CBE and mammogram screening, BSE, communication skills, how to address potential barriers and the importance of confidentiality. CHWs will practice their skills during role play using a prepared calling script to guide the phone conversation with participants. Two male volunteers will also participate in the CHW training and learn to speak to the participant's husbands or male relatives about participating in the study, if requested. Telephone conversations between the CHWs and participants will be recorded as per protocol. CHWs will receive a monthly phone credit and a one-off cash allowance for their time.

Comparator Group

During the recruitment call, participants from the CG will be made aware that BC is the commonest cancer amongst women in Malaysia and about the importance of early detection. Women who will be randomized to the CG will not receive the intervention but may avail of 'usual' screening via their local clinic (subject to pandemic restrictions).

Pilot study

We will recruit 40-50 participants to test the intervention in September 2021. Trained SEACO research staff and CHWs will be asked to record the pilot phone calls. The research team will provide feedback and evidence-informed further training to the research staff and CHWs based on the pilot calls. All pilot participants will be surveyed and the results will inform potential modifications and further training requirements for the CHWs.

Evaluation

Outcome measurements

Our primary measure is the difference in CBE screening uptake between women who receive the CHW-led education and navigation intervention and women from the CG who receive usual standard practice (i.e. opportunistic screening at clinics) during the pandemic. In addition to increased awareness about the CBE screening and engagement with the screening programme, secondary outcomes include positive change related to:

- BC symptom recognition
- Intention to attend CBE and mammogram screening
- Beliefs and barriers regarding BC and BC screening
- Practice of breast-self-examination (BSE)
- Mammogram screening attendance (for CBE positive women)

Outcomes related to the programme evaluation are informed by the RE-AIM framework, i.e. reach, effectiveness, adoption, implementation and maintenance [39], together with measures of acceptability, appropriateness and feasibility [40].

Methods of assessment

Participant information

Information about gender, age, ethnicity, income, education, occupation, having somatic NCDs (e.g. diabetes and hypertension), body mass index (BMI), smoking status, cancer history, travel distance (km) to the nearest clinic and ownership of motorised vehicle will be extracted from the most recent health survey (2018) recorded in the SEACO database in order to present a profile of study participants. Participants will be asked questions about mobile phone ownership/usage and internet usage during the baseline survey.

Surveys (baseline and follow-up)

The baseline and follow-up surveys will be completed over the phone. Trained SEACO data collectors will conduct telephone surveys with participants from the IG and CG that will take approximately 15-20 minutes. The follow-up survey will take between 20-25 min for the IG and 15-20 min for the CG. The surveys comprise a number of previously adapted and validated questionnaires. In order to ascertain changes in BC symptom recognition, we will include the validated Breast Cancer Awareness Measure for Malaysia (B-CAM-M) [41] before-and-after the intervention implementation. CBE and mammogram screening intentions at baseline will be measured by asking participants to respond to the statement ‘I intend to have a Clinical Breast Examination to check for breast cancer in the near future’, using a 5-point Likert scale (strongly disagree – strongly agree), providing the option ‘I have not yet

thought about this'. Unless participants choose the last option, participants will also be asked at what age they are thinking of getting the CBE [42]. The same questions will be asked about participant's intention to attend mammogram screening. The follow-up survey will also include specific questions to assess participant satisfaction with the screening [43,44] as well as the acceptability and appropriateness of the intervention based on an adapted version of the validated Acceptability E-scale [45] (IG only).

BC screening attendance

LPPKN nurses will be asked to record CBE attendance in a spreadsheet that will be shared with SEACO on a weekly basis. Mammogram attendance will be collected retrospectively from the LPPKN clinic.

Statistical analysis

Quantitative data will be analysed with SPSS vs 24. Descriptive statistics at baseline will be reported as mean (SD) for continuous data and frequencies (percentages) for categorical data. The change in screening uptake and other outcomes will be compared between the IG and CG using student independent samples t-test and Chi-square tests. A Chi-square test will compare the proportion of IG and CG participants who attended CBE after completion of intervention delivery, approximately 4-6 weeks after completing the survey. The difference in proportion or odds ratio or relative risk (and 95% CI) will be used as an initial indictor of effect size and we will aim to estimate the 'number needed to treat' in terms of the number of patients that would need to be given the intervention for one extra to be screened (via CBE). Change within groups will be analysed using paired samples t-test for continuous variables and Chi-square test for categorical variables. Multivariate regression models will be used to investigate factors (e.g. ethnicity, age, income, marital status, education, BC history and BC screening history) affecting screening participation.

Sampling size and procedure

Women from Sungai Segamat and Jabi will be randomized to either IG (n=466) or CG (n=466) prior to recruitment. A sample of 1600 women (n=800 for the IG and CG) will be drawn from the SEACO database to account for participants who are not interested in participating. A sample size of 932 will allow 80% power to detect, as statistically significant at the 5% level, an increase of 9% or more in the proportion aware of a BC symptom in the IG compared to the CG after the intervention (based upon baseline awareness of a breast lump as a cancer symptom of 65% from our previous Newton-Ungku-Omar funded study). The sample size of 932 will also allow 80% power to detect an absolute increase in the proportion of women who avail of a CBE following receipt of the intervention of the intervention of 9% compared with the comparator group. The pilot will be tested with 40-50 participants.

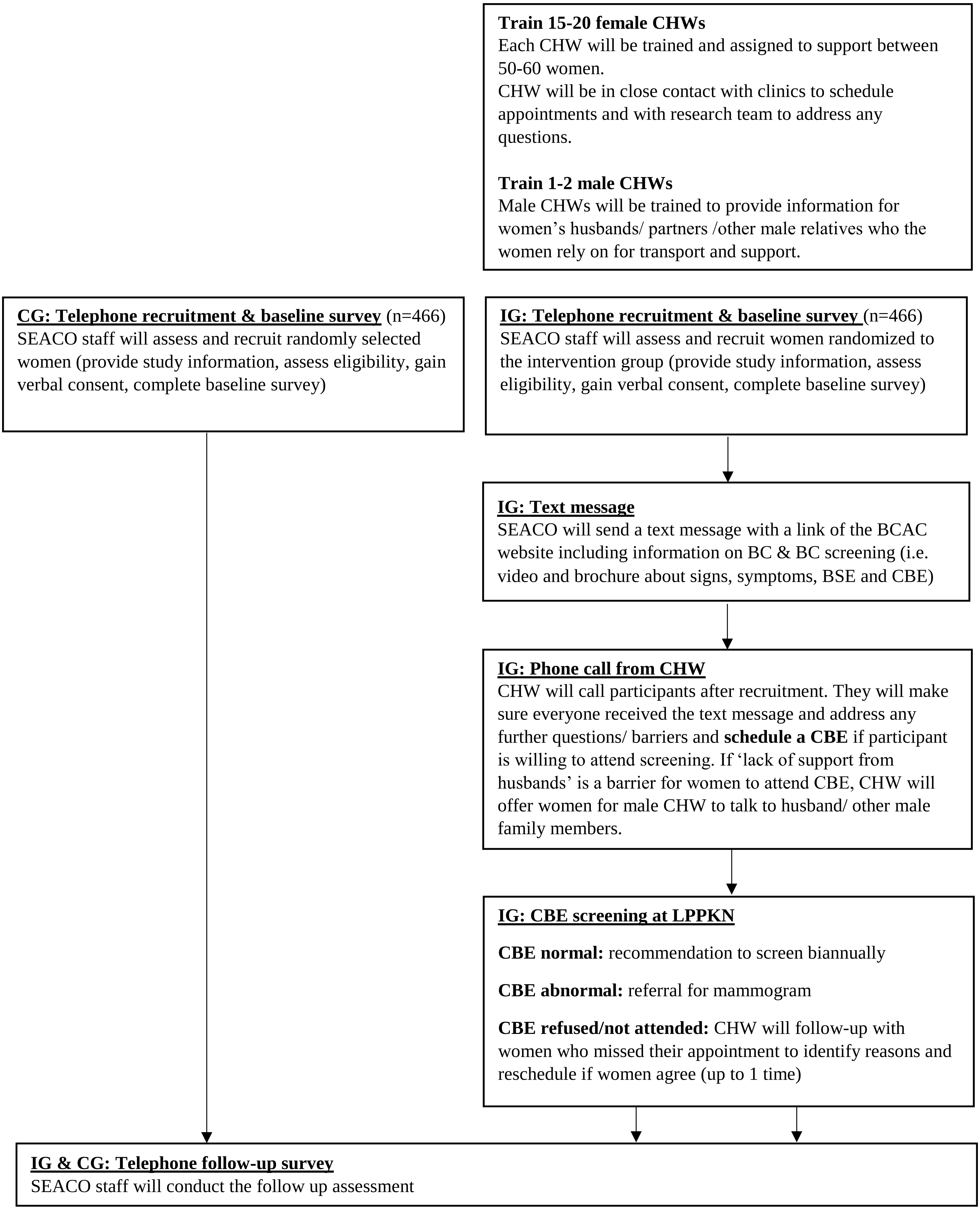

Figure 1. CENP study flow

Table 1 Barriers to BC early detection mapped onto the subcomponents of the COM-B model and how the CENP intervention aims to address those (look at https://implementationscience.biomedcentral.com/track/pdf/10.1186/s13012-018-0821-y.pdf)

	**Capability**		**Opportunity**		**Motivation**	
	**Physical**	**Psychological**	**Physical**	**Social**	**Reflective**	**Automatic**
**System level**	Limited mammogram facilities		Movement Control Order	Cultural norms<br>Language barriers	Number of subsidised mammograms and quote for screening is relatively low	Absence of system to record/ remind doctors when to screen
**Provider level**	Varying CBE skills	Lack of awareness about breast cancer and screening among non-specialists	Time constraints<br>Understaffed clinics	Provider-patient communication<br>Dominantly male doctors		BC screening is not prioritised
**Patient level**	Lack of BSE practice skills	Lack of awareness about BC and importance of early detection	Cost to patient<br>Transport to clinics<br>Long waiting times at clinic	Husband are not always supportive	Low perceived susceptibility	Embarrassment<br>Fear
**CENP Intervention**<br>Opportunities mapped onto the subcomponents of the COM-B model	LPPKN clinic has experienced nurses trained in CBE.<br>Participants will be referred to the BCAC website for information on BSE by the CHWs.	LPPKN clinic has experienced nurses trained in CBE.<br>Participants will be referred to the BCAC information website and will received information from the CHWs.	CBE, mammogram and transport costs will be covered for participants.<br>Appointment scheduling to reduce waiting times and ensure staff availability	Trained CHWs will be matched with participants based on ethnic and language background.<br>Male CHWs will be trained to support husbands.<br>LPPKN nurses are all females.	Participants will be educated on BC risk and susceptibility by the CHWs.	Barriers such as embarrassment and fear will be addressed by the CHWs prior to screening.

Abbreviations

BC –	Breast Cancer
BCAC –	Be Cancer Alert Campaign
BCSS -	Breast Cancer Support Society Segamat
CBE –	Clinical Breast Examination
CG –	Comparator Group
CHW –	Community Health Worker
IG –	Intervention Group
LPPKN -	Lembaga Penduduk dan Pembangunan Keluarga Negara (translation: National Population and Family Development Board Malaysia)
mHealth –	mobile Health
SEACO -	South East Asian Community Observatory
WHO -	World Health Organization

Declarations

Declaration of conflicting interests: The authors declare that they have no competing interest.

Funding: This study is funded by MRC UK (Ref: 537084059) and MIGHT (Ref: 2500235-122-00). The grant application was subject to peer-review by individual academic reviewers and the final decision about funding was made by an expert panel.

Research ethics and patient consent: Ethics approval and consent to participate Ethics approval has been granted by the Monash University Human Research Ethics Committee (ID: 29682). Participants will be asked to provide verbal consent prior to each survey.

Acknowledgements: We would like to thank nurses from the LPPKN clinic in Segamat for facilitating CBE and mammograms for this study and all study participants involved in the qualitative and quantitative aspects of the evidence synthesis for their contribution.

References

1. Ministry of Health Malaysia. Malaysia National Cancer Registry Report 2012-2016. Putrajaya; 2020.

2. International Agency for Research on Cancer. GLOBOCAN [Internet]. 2018. Available from: http://gco.iarc.fr/today/home

3. Bhoo Pathy N, Verkooijen HM, Taib NA, Lee SC, Saxena N, Iau P, et al. Association between ethnicity and survival after breast cancer in a multi-ethnic Asian setting: Results from the Singapore-Malaysia hospital-based breast cancer registry. J Univ Malaya Med Cent. 2013;16:90.

4. Cheng ML, Ling DY, Nanu PKP, Nording H, Lim CH. Factors influencing late stage of breast cancer at presentation in a district Hospital - Segamat Hospital, Johor. Med J Malaysia. 2015;70:148–52.

5. World Health Organization. Breast cancer: prevention and control [Internet]. 2021 [cited 2021 May 19]. Available from: https://www.who.int/cancer/detection/breastcancer/en/

6. Anderson BO, Ilbawi AM, Fidarova E, Weiderpass E, Stevens L, Abdel-Wahab M, et al. The Global Breast Cancer Initiative: a strategic collaboration to strengthen health care for non-communicable diseases. Lancet Oncol. 2021;22:578–81.

7. Ngan TT, Nguyen NTQ, Van Minh H, Donnelly M, O'Neill C. Effectiveness of clinical breast examination as a 'stand-alone' screening modality: an overview of systematic reviews. BMC Cancer. BMC Cancer; 2020;20:1–10.

8. Htay MNN, Donnelly M, Schliemann D, Yim S, Dahlui M, Somasundaram S, et al. Breast Cancer Screening in Malaysia : A Policy Review. Asian Pacific J Cancer Prev. 2021;22:1685–93.

9. Farid NDN, Aziz NA, Al-Sadat N, Jamaludin M, Dahlui M. Clinical breast examination as the recommended breast cancer screening modality in a rural community in Malaysia; what are the factors that could enhance its uptake? PLoS One. 2014;9:1–6.

10. Schliemann D, Htay MNN, Dahlui M, Paramasivam D, Cardwell CR, Ibrahim Tamin NSB, et al. Impact of a mass media campaign on breast cancer symptoms awareness and screening uptake in Malaysia: findings from a quasi-experimental study. BMJ Open. 2020;10:e036503.

11. Jones CEL, Maben J, Jack RH, Davies EA, Forbes LJL, Lucas G, et al. A systematic review of barriers to early presentation and diagnosis with breast cancer among black women. BMJ Open. 2014;4.

12. Schliemann D, Ismail R, Donnelly M, Su TT. Anticipated delay in help-seeking for cancer symptoms: Findings from a nationwide study of Malaysian adults. Cancer Epidemiol. 2021;71.

13. O'Donovan J, O'Donovan C, Nagraj S. The role of community health workers in cervical cancer screening in low-income and middle-income countries: A systematic scoping review of the literature. BMJ Glob Heal. 2019;4:1–8.

14. Hand T, Rosseau NA, Stiles CE, Sheih T, Oluwasanu M, Olopade OI, et al. The global role, impact, and limitations of Community Health Workers (CHWs) in breast cancer screening: a scoping review and recommendations to promote health equity for all ABSTRACT. Glob Health Action [Internet]. Taylor & Francis; 2021;14. Available from: https://doi.org/10.1080/16549716.2021.1883336

15. Ballard M, Bancroft E, Nesbit J, Johnson A, Holeman I, Foth J, et al. Prioritising the role of community health workers in the COVID-19 response. BMJ Glob Heal. 2020;1–7.

16. Asadzadeh A, Kalankesh LR. A scope of mobile health solutions in COVID-19 pandemics. Informatics Med Unlocked [Internet]. Elsevier Ltd; 2021;23:100558. Available from: https://doi.org/10.1016/j.imu.2021.100558

17. Budd J, Miller BS, Manning EM, Lampos V, Zhuang M, Edelstein M, et al. Digital technologies in the public-health response to COVID-19. Nat Med [Internet]. Springer US; 2020;26:1183–92. Available from: http://dx.doi.org/10.1038/s41591-020-1011-4

18. Kataria I, Ngongo C, Lim SC, Kocher E, Kowal P, Chandran A, et al. Development and evaluation of a digital, community-based intervention to reduce noncommunicable disease risk in a low-resource urban setting in Malaysia: a research protocol. Implement Sci Commun. Implementation Science Communications; 2020;1:1–9.

19. World Health Organisation. Global Strategy on Digital Health. 2020.

20. United Nations. Sustainable Development Goals [Internet]. 2015 [cited 2021 Jun 11]. Available from: https://sdgs.un.org/goals

21. Basu P, Alhomoud S, Taghavi K, Carvalho AL, Lucas E. Cancer Screening in the Coronavirus Pandemic Era : Adjusting to a New Situation special articles abstract. JCO Glob Oncol. 2021;

22. Cancino RS, Su Z, Mesa R, Tomlinson GE. The Impact of COVID-19 on Cancer Screening : Challenges and Opportunities Corresponding Author : JMIR Cancer. 2020;6:1–13.

23. Craig P, Dieppe P, Macintyre S, Michie S, Nazareth I, Petticrew M. Developing and evaluating complex interventions: the new Medical Research Council guidance. BMJ. 2008;337:a1655.

24. Smits S, Mccutchan G, Wood F, Edwards A, Lewis I, Robling M, et al. Development of a Behavior Change Intervention to Encourage Timely Cancer Symptom Presentation Among People Living in Deprived Communities Using the Behavior Change Wheel Using the Behavior Change

Wheel. Ann Behav Med. 2018;474–88.

25. Surgey A, Disbeschl S, Lewis R, Hiscock J, Nafees S, Law RJ, et al. ThinkCancer! The multi-method development of a complex behaviour change intervention to improve the early diagnosis of cancer in primary care. medRxiv [Internet]. 2021; Available from: http://medrxiv.org/content/early/2021/06/24/2020.11.20.20235614.abstract

26. Michie S, Stralen MM Van, West R. The behaviour change wheel : A new method for characterising and designing behaviour change interventions. Implement Sci. 2011;6.

27. Michie S, van Stralen MM, West R. The behaviour change wheel: A new method for characterising and designing behaviour change interventions. Implement Sci. 2011;6.

28. Tan MM, Schliemann D, Hoe Mok WK, Mohan D, Taib NAM, Donnelly M, et al. mHealth interventions to improve cancer screening and early detection: A scoping review of reviews. (submitted).

29. Schliemann D, Kok Hoe WM, Mohan D, Allotey P, Reidpath DD, Taib NAM, et al. Challenges and opportunities for breast cancer early detection among rural dwelling women in Malaysia: a qualitative study. (submitted).

30. Mohan D, Su TT, Donnelly M, Kok Hoe WM, Schliemann D. Breast Cancer Screening in Semi-Rural Malaysia: Utilisation and Barriers. Int J Environ Res Public Health. 2021;18:1–14.

31. Department of Statistics Malaysia. Current Population Estimates Malaysia 2020 [Internet]. 2020 [cited 2021 Jun 11]. Available from: https://www.dosm.gov.my/v1/index.php?r=column/cthemeByCat&cat=155&bul_id=OVByWjg5YkQ3MWFZRTN5bDJiaEVhZz09&menu_id=L0pheU43NWJwRWVSZklWdzQ4TlhUUT09

32. Statista. Malaysia: Urbanization from 2009 to 2019 [Internet]. 2021 [cited 2021 Jun 11]. Available from: https://www.statista.com/statistics/455880/urbanization-in-malaysia/

33. Partap U, Young EH, Allotey P, Soyiri IN, Jahan N, Komahan K, et al. HDSS Profile: The South East Asia Community Observatory Health and Demographic Surveillance System (SEACO HDSS). Int J Epidemiol [Internet]. 2017;46:1370-1371g. Available from: http://academic.oup.com/ije/article/46/5/1370/4037470

34. Kennedy C, Mann CB. RANDOMIZE: Stata module to create random assignments for experimental trials, including blocking, balance checking, and automated randomization. Boston College Department of Economics; 2015.

35. O'Donovan J, Newcomb A, Macrae MC, Vieira D, Onyilofor C, Ginsburg O. Community health workers and early detection of breast cancer in low- - income countries: a systematic scoping review

of the literature. BMJ Glob Heal. 2020;5.

36. Monash University Malaysia, Queen's University Belfast, University of Malaya, National Cancer Society Malaysia. Be Cancer Alert: Our Campaign [Internet]. 2018 [cited 2021 Aug 24]. Available from: http://www.becanceralert.com/breast-cancer/our-campaign/

37. Ministry of Health Malaysia. Management of Breast Cancer. Clin Pract Guidel [Internet]. 2010;1–100. Available from: http://www.degruyter.com/view/books/9783050047348/9783050047348.123/9783050047348.123.xml

38. Wells KJ, Luque JS, Mildanovic B, Vargas N, Asvat Y, Roetzheim RG, et al. Do Community Health Worker Interventions Improve Rates of Screening Mammography in the United States? A Systematic Review. Cancer Epidemiol Biomarkers Prev. 2011;20:1580–98.

39. Dzewaltowski DA, Glasgow RE, Klesges LM, Estabrooks PA, Brock E. RE-AIM: evidence-based standards and a Web resource to improve translation of research into practice. Ann Behav Med. 2004;28:75–80.

40. Proctor E, Silmere H, Raghavan R, Hovmand P, Aarons G, Bunger A, et al. Outcomes for implementation research: Conceptual distinctions, measurement challenges, and research agenda. Adm Policy Ment Health. 2011;38:65–76.

41. Htay MNN, Donnelly M, Schliemann D, Loh SY, Dahlui M, Ibrahim Tamin NSB, et al. Translation and validation of the breast cancer awareness measurement tool in Malaysia (B-CAM-M). Asian Pacific J Cancer Prev. 2020;21:217–23.

42. Labrie NHM, Ludolph R, Schulz PJ. Investigating young women's motivations to engage in early mammography screening in Switzerland: Results of a cross-sectional study. BMC Cancer. BMC Cancer; 2017;17:1–10.

43. Iversen HH, Holmboe O, Bjertnæs ØA. The Cancer Patient Experiences Questionnaire (CPEQ): reliability and construct validity following a national survey to assess hospital cancer care from the patient perspective. BMJ Open. 2012;

44. Yoon NH, Lee HY, Kwak MS, Choi KS, Jun JK, Kim MK, et al. Comparison of satisfaction with cancer screening at mobile van and static sites: National cancer screening program in Korea. Jpn J Clin Oncol. 2009;39:169–74.

45. Tariman JD, Berry DL, Halpenny B, Wolpin S, Schepp K. Validation and testing of the Acceptability E-scale for Web-based patient-reported outcomes in cancer care. Appl Nurs Res [Internet]. 2011;24:53–8. Available from:

https://www.ncbi.nlm.nih.gov/pmc/articles/PMC3624763/pdf/nihms412728.pdf
