# Supplementary material for: The development and evaluation of a mHealth, community education and navigation intervention to improve clinical breast examination uptake in Segamat Malaysia: A randomised controlled trial
Source: PLoS One. 2023 Oct 5;18(10):e0288437. doi: 10.1371/journal.pone.0288437 (PMC10553222; doi:10.1371/journal.pone.0288437)
Supplement: S3 File — (PDF) [file pone.0288437.s004.pdf]

Study record 42195

Generated: 09/08/2022 13:09:51
Editorial Status: Ready for publication

Title and Additional Identifiers

Submission number
42195

ISRCTN

DOI

Public title
Community education and navigation to improve breast cancer screening uptake in Malaysia

Scientific title
Improving early detection of breast cancer in Malaysia during the COVID-19 pandemic - the use of mHealth to improve community education and navigation: a study protocol

Acronym
CENP

EudraCT number
Nil known

IRAS number

ClinicalTrials.gov number
Nil known

Protocol /serial number
537084059

Condition category
Cancer

Date Applied
08/08/2022

Date Assigned

Last Edited
09/08/2022

Prospective/Retrospective

Overall Trial Status

Completed

Recruitment status
No longer recruiting

Study Information

Study hypothesis
Our main aim was to design, implement and evaluate an intervention to improve uptake of Clinical Breast Examination (CBE) screening in Malaysia and breast cancer symptom recognition in the context of the COVID-19 pandemic.

Ethics approval
Approved 01/09/2021, Monash University Human Research Ethics Committee (Monash University, Malaysia; muhrec@monash.edu; +61 3 990 52052), ref: 29682

Study design
Interventional randomized controlled trial

Primary study design
Interventional

Secondary study design
Randomised controlled trial

Trial setting
Community

Trial type
Screening

Overall trial start date
03/01/2021

Overall trial end date
31/03/2022

Overall trial status override

Reason abandoned (if study stopped)

Condition
Clinical Breast Examination (CBE) screening for breast cancer early detection

Interventions
This study was a randomised controlled trial and participants were randomised to either an intervention group (IG) or a control group (CG). Participant enrolment was conducted by trained data collectors. All women who were randomised to the IG or CG were invited to participate by trained data collectors over the phone. The resources for this study did not permit separation of enrollment and data collection procedures and, so, data collectors were aware of the group to

which women were assigned. Participants and data collectors were not blinded during the enrollment and surveys.

The IG received a multi-component mHealth intervention i.e. information about breast cancer was provided through an educational website, and telephone calls and text messages from community health workers (CHWs) raised breast cancer awareness and offered to navigate women to CBE services. The usual free opportunistic screening service was available for CG participants. It was not possible to 'blind' participants, data collectors and CHWs.

Intervention Type
Behavioural

Phase

Drug name(s)

Primary outcome measure
Clinical breast examination (CBE) screening uptake measured using records from the LPPKN clinic at follow-up as well as self-reported data at baseline and follow-up.

Secondary outcome measures
1. Breast cancer symptom recognition measured using the Breast Cancer Awareness Measure for Malaysia (B-CAM-M) at baseline and follow-up.
2. Intention to attend a CBE measured using the question 'I intend to have a Clinical Breast Examination to check for breast cancer in the near future' and a 5-point Likert scale for participants to rate their response at baseline and follow-up.
3. Beliefs and barriers regarding breast cancer and breast cancer screening measured using the Breast Cancer Awareness Measure for Malaysia (B-CAM-M) at baseline and follow-up.
4. Practice of breast self-examination measured using the Breast Cancer Awareness Measure for Malaysia (B-CAM-M) at baseline and follow-up.
5. Mammogram screening attendance (for CBE-positive women) measured using LPPKN clinic records at follow-up.

Trial website

Participant information sheet
Not available in web format, please use contact details to request a participant information sheet.

Eligibility

Participant inclusion criteria
1. Residents from Sungai Segamat and Jabi (Malaysia) who were recorded in the South East Asia Community Observatory (SEACO) database, completed the 2018 SEACO health survey and have previously given consent to be contacted about participating in other research studies.
2. Women aged 40 - 74 years
3. Registered a phone number with SEACO. Only women who were able to provide a mobile phone number when contacted were able to participate in the study.

Participant type
Healthy volunteer

Age group
Adult

Gender
Female

Target number of participants
932

Total final Enrolment
483

Participant exclusion criteria
1. Breast cancer patients and survivors.
2. Women who reported to the researchers at the time of the interview that they are experiencing breast cancer symptoms were excluded from the research and encouraged to seek help from their local doctor as soon as possible.

Recruitment start date
02/09/2021

Recruitment end date
14/11/2021

Recruitment status override

Locations

Countries of recruitment
Malaysia

Trial participating centres

Trial Centre

Trial Centre Name
South East Asia Community Observatory

Address
125, Jalan Sia Her Yam
Kampung Abdullah

City
Segamat District, Johor

Country
Malaysia

Zip
85000

Trial Centre

Trial Centre Name
Klinik LPPKN Segamat

Address
Kawasan Hospital Segamat
Jalan Muar

City
Segamat District, Johor

Country
Malaysia

Zip
85000

Plain English Summary

Background and study aims
Breast cancer screening uptake in Malaysia is low and a high number of cases present at a late stage. Community navigation and mobile health (mHealth) may increase screening attendance, particularly by women from rural communities. This randomized controlled study evaluated an intervention that used mHealth and community health workers to educate women about breast cancer screening (i.e. clinical breast examination - CBE) and navigate them to CBE services in the context of the COVID-19 pandemic.

Who can participate?
The target population comprised residents from Sungai Segamat and Jabi who were recorded in the South East Asia Community Observatory (SEACO) database, completed the SEACO health survey in 2018, and, previously, gave consent to be contacted about participating in research studies. Thus, we contacted women aged 40-74 years who had registered their phone number with SEACO. Only women who had a mobile phone number via which they could be contacted were able to participate in the study. BC patients and survivors, and women who reported to the research team at the time of the interview that they were experiencing BC symptoms were excluded from the research and encouraged to seek help from their local doctor as soon as possible.

What does the study involve?
Women from the two sub-districts in the SEACO database were randomized to the intervention group (IG) or comparator group (CG) with a 1:1 allocation prior to being contacted. Women who were randomized to the IG received an intervention consisting of mHealth education and community navigation by community health workers (CHWs). CHWs called the women to whom they had been assigned during the same week that the baseline assessment interview had been completed to discuss breast cancer symptoms and breast self-examination, address barriers to

screening, and ask them if they were interested in attending a CBE at the LPPKN clinic in Segamat. CBE appointments were arranged to occur the following week at a time that suited interested participants. CHWs discussed with participants who did not want to avail of the offer of screening their concerns and fears, but respected the decision of women who reiterated that they did not want to attend the CBE. Women with normal CBE results were asked by LPPKN nurses to attend screening biannually, either at the LPPKN or at a health clinic, as recommended in the clinical practice guidelines. A doctor at the LPPKN clinic met with women who received an abnormal CBE finding and referred them for a mammogram at the hospital as soon as possible after their CBE, free of charge.

The SEACO-trained data collectors contacted (via phone) women who were randomised to the CG - during this call, they informed women who agreed to participate that BC is the most common cancer amongst women in Malaysia and about the importance of early detection. Women in the CG did not receive the intervention (described above) but they could avail of 'usual' screening via their local clinic – however, elective procedures were stopped at the local government clinics to facilitate COVID-19 vaccinations and patients. In addition, CG participants were offered a scheduled free CBE at the LPPKN clinic after the follow-up data collection phase had been completed.

Information about gender, age, ethnicity, household income, marital status, education, occupation and study sub-district were extracted from the most recent health survey (2018) recorded in the SEACO database in order to present a profile of study participants. Participants were asked questions about mobile phone ownership/usage and internet usage during a baseline telephone survey. baseline and follow-up survey interviews were completed over the phone. Trained SEACO data collectors conducted telephone interviews with participants from the IG and CG that took approximately 15-20 minutes. The follow-up survey took between 20-25 min for the IG and 15-20 min for the CG. The survey interview comprised a number of previously adapted and validated questionnaires. LPPKN nurses were asked to record CBE attendance in a spreadsheet that was shared with SEACO on a weekly basis.

What are the possible benefits and risks of participating?
There were no immediate and direct benefits to participants; but the collected information will help to identify factors that may affect public awareness of breast cancer. Discussing and recollecting unpleasant feelings, fears, or worries about a sensitive topic such as breast cancer may have caused discomfort and distress. If participants felt upset at any stages of the study and wanted to talk about their concerns, we provided them with contact details of the Chief Investigator or encouraged them to call the National Cancer Society Malaysia hotline at 1800-88-1000. We do not expect any long-term risks associated with participating in a clinical breast examination.

Where is the study run from?
Queen's University Belfast (UK)

When is the study starting and how long is it expected to run for?
January 2021 to March 2022

Who is funding the study?
Medical Research Council and Newton (UK) (Ref: 537084059) funded the project and the PI of the funding received was Prof Michael Donnelly.
Malaysian Industry-Government Group for High Technology (MIGHT) (Ref: 2500235-122-00) funded Prof Tin Tin Su as Principal Investigator on this project.

Who is the main contact?
Prof. Michael Donnelly, michael.donnelly@qub.ac.uk
Prof. Tin Tin Su, TinTin.Su@monash.edu

Results and Publications

Publication and dissemination plan
The findings of our study will be disseminated to LMICs through publications and presentations at conferences as well as to advocacy groups and stakeholder groups and professional networks in Malaysia.

IPD Sharing statement
The datasets generated during and/or analysed during the current study are/will be available upon request from (South East Asia Community Observatory; mum.seaco@monash.edu; quantitative data; available from 01/01/2023 until 01/01/2028; information on how to request data can be found here: https://www.monash.edu.my/seaco/research-and-training/how-to-collaborate-with-seaco; participants consented for their data to be analysed anonymously for research purposes, identifying information will kept strictly confidential by only using SEACO ID numbers).

Intention to publish date
01/10/2022

Participant level data
Available on request

Basic results (scientific)

Results (plain English)

Publication list

Publication citation(s)

Contact(s)

Contact

Type
Principal Investigator

Title
Prof

Name
Michael Donnelly

ORCID ID

http://orcid.org/0000-0001-8380-7413

Address
Centre for Public Health
Institute of Clinical Sciences
Block B, Royal Victory Hospital
Grosvenor Road

City
Belfast

Country
United Kingdom

Zip
BT12 6BA

Tel
+44 2890978993

Email
michael.donnelly@qub.ac.uk

Privacy
Public

Contact

Type
Principal Investigator

Title
Prof

Name
Tin Tin Su

ORCID ID
http://orcid.org/0000-0003-0387-6406

Address
Monash University Malaysia
Jeffrey Cheah School of Medicine & Health Sciences
Jalan Lagoon Selatan
Bandar Sunway

City
Subang Jaya

Country
Malaysia

Zip
47500

Tel
+603-5514 4472

Email
tintin.su@monash.edu

Privacy
Public

Sponsor(s)

Sponsor

Organisation
Queen's University Belfast

Address
University Road

City
Belfast

Country
United Kingdom

Zip
BT7 1NN

Tel
+44 28 9024 5133

Email
cph@qub.ac.uk

Type
University/education

Website
http://www.qub.ac.uk/

Privacy
Public

Funder(s)

Funding Type
Research council

Funder

Funder Name
Medical Research Council

Alternative Name(s)
UK Medical Research Council MRC

Funding Body Type
Government organisation

Funding Body Subtype
National government

Location
United Kingdom

Funder

Funder Name
Malaysian Industry-Government Group for High Technology (MIGHT)

Alternative Name(s)

Funding Body Type

Funding Body Subtype

Location

Applicant Details

Name
Desiree Schliemann

ORCID ID

Address

City

Country

Zip

Tel

Email
d.schliemann@qub.ac.uk

Payment Method

Payment method
Online payment

Trusted funder

Invoice Details

Name

Institution

Address

City

State

Country

Zip

Email

Purchase order/ reference number

VAT number

Why did you choose ISRCTN to register your trial?
Straightforward registration process
