# Supplementary material for: The development and evaluation of a mHealth, community education and navigation intervention to improve clinical breast examination uptake in Segamat Malaysia: A randomised controlled trial
Source: PLoS One. 2023 Oct 5;18(10):e0288437. doi: 10.1371/journal.pone.0288437 (PMC10553222; doi:10.1371/journal.pone.0288437)
Supplement: Attachment — Submitted filename: comments.docx [file pone.0288437.s005.docx]

**Reviewer-1**

General comments:

Breast cancer is a threat to women especially in Low and Middle Income Countries (LMICs) as presented by the authors. Interventions aimed at preventing and treating breast cancer are encouraged. Therefore, this manuscript is addressing an important topic. In general, the manuscript is well written but due to the small sample size did not meet the required strength to detect differences between the two groups, intervention and comparator. The authors need to recalculate the resulting detection power from the achieved sample size.

Specific comments

Introduction

1. Article 1 is over referenced in the first paragraph

2. Under line 84, I suggest using the word, “participants” instead of the, “sample”.

3. Line 90, “These factors explain why BC patients…”

4. Line 102, do you mean that travel restrictions resulted to an increase of Breast Cancer cases?

Methods

Study population

1. Line 137, only women who had a mobile phone number … were considered for participation. If it is true that mobile phone ownership is an indication of a socioeconomic status, especially in LMICs, then this criterion introduced a bias that the authors should cite as a limitation. Also, it is important to provide information about the proportion of the population that owns a mobile phone.

2. Line 178… A one-off reimbursement for transportation fees to the clinic was offered to participants. How much was the transport fees, and was it enough to influence participants’ decision?

Comparator

1. Line 208, elective procedures were stopped at the local government clinics to facilitate COVID-19 vaccinations and patients. Could this act minimize alternatives and hence increase demand for the services from the study clinic? Meaning, the demand for CBE was not only due to the intervention. Unfortunately, there are no data from the comparator group on CBE.

2. Consider providing more information, for example, the purpose for BC screening attendance (Line 249) and Qualitative interviews (Line 253).

Sample size and procedures

1. Line 270, align information about sample size with that provided in Figure 1. For example, at what stage were participants allocated to the two arms?

2. Line 277, indicates that a pilot study was conducted. If the study findings were published, then I suggest referencing the article. However, if the pilot study findings have not been published, then the authors should consider providing a summary of the pilot study as part of the study methods.

Results

1. Line 288, consider providing conversion rate of the currency to US dollar.

2. Line 306, Instead of using the term, “Single” to mean those who have never married, separated/divorced and widowed, you could use terms like, “Has a partner/No partner”

**Reviewer-2**

Abstract
- CBE is mentioned in the introduction of the abstract without being stated in full (as has been done with BC and mHealth)
- the methods of the abstract says 'regression analyses were conducted to investigate between-group differences over time...'; it would be better to use this opportunity to state what the outcomes were, i.e. 'between group differences in uptake of screening and attendance of CBE...'
- the results in the abstract are confusing, perhaps because they don't focus or highlight on the overall effect of the intervention on the main outcome(s) of the trial. Why do they mention household income at all? This results section should first describe the effect of the intervention on the main outcomes, and if there is anything further to say about subgroups then those can be subsequently mentioned; otherwise it may look like the authors are cherry-picking 'significant' results to report.

Introduction
- it seems a little out of place to mention the statistics on the rates of all cancers in males and females between two sentences talking about breast cancer (lines 63-65 and 65-67) - unless these lines are also referring to breast cancer, in which case this needs to be written more clearly.
- the statement in line 159-161 about the CONSORT diagram is a result and should be moved to the results section.

Methods
- in line 215, what you probably mean to say is that your primary measure was CBE screening uptake (perhaps more clearly, the proportion of women to took up CBE screening); the 'difference' is not an outcome but a measure of effect. Similarly, in line 219 your secondary outcomes are self-reported intention to attend CBE and mammogram screening - saying 'nature and degree...' is non-specific language in what should be unambiguous.
- in line 220, there is need for further clarity i.e. to state explicitly which outcomes were informed by the RE-AIM framework.
- reading lines 232-247, it is starting to seem to me that the primary outcome of CBE uptake was a categorical (perhaps ordered categorical) variable, although I can also see how a binary outcome could have been derived out of it. And the only reason I am thinking about a binary outcome is the previous wording of how this outcome has been described and also the mention of the proportions of CBE uptake in the abstract. What I am getting at here is that the actual outcome is not clearly described anywhere. If a binary outcome was derived from the likert scale, this needs to be clearly described. If the likert scale was used as is, this also needs to be clearly described (and if this is what was done then some aspects of the results don't make sense).
- if the primary outcome was binary, then although you could use chi-squared tests for a basic analysis, it would be better to use logistic regression models, if only so that you can present unadjusted and adjusted estimates of effect. In this case, chi-squared tests would be unnecessary. If the primary outcome was categorical, then this approach is fine, although you could also use multinomial regression models to obtain unadjusted and adjusted effects. If the outcome is ordered categorical, then you could use ordinal logistic regressions to obtain unadjusted and adjusted effect. Again, given the lack of clarity on exactly what the primary outcome is, I am unable to determine which of these I would recommend.
- again, for continuous outcomes, there is no need to use t-tests when you could use linear regression models to obtain unadjusted and adjusted effects. Here you don't need the change-in-screening intention score: you should simply regress the endpoint scores on treatment group (and any covariates, in the adjusted model) further adjusting for the baseline scores; this is an analysis of covariance model, which is the most statistically efficient approach for a continuous outcome with a baseline measure (and which gives identical estimates to a regression of change-from-baseline scores adjusting for baseline, given that baseline adjustment is pertinent for the improved precision of effect estimates).
- the analysis exploring factors affecting screening participation is a secondary question and should not be conflated with that seeking to estimate the effect of the intervention.
- I was not able to reproduce your sample size calculation. In my calculations, you would need about 410 participants in each arm (i.e. 820 total for two arms with 1:1 allocation) to detect a 9% absolute improvement in a binary outcome assuming a baseline level of 65%, with 80% power at the 5% level of significance for a two-tailed test. Your calculation also mentions some adjustment of recruitment to account for attrition; you would normally indicate your anticipated level of attrition as this would normally be included in the calculation
- regarding adjustments, you would normally adjust for age (and sex if relevant, but may not be relevant here if your sample was all/mostly female) and any baseline factors relevant during randomisation.

Results
- the way the results have been presented distract from the objectives of the study, in my opinion. Since the study aimed to investigate the effect of the intervention, the results should have began with a summary of the CONSORT flow diagram i.e. the numbers of participants in the treatment arms that were eligible, screened, randomised, followed-up, and for whom outcomes were available. This should have then been followed up by a table of the baseline characteristics of participants with outcomes to be included in the main analysis, split by treatment group. Crucially, this table must not include statistical tests/p-values comparing the treatment arms with respect to baseline characteristics, because in a randomised trial it is expected that the groups would be balanced and any imbalance is random and not particularly statistically interesting. Thus, the p-values in Table 3 are unnecessary, as are the separate columns for 'baseline' and 'follow-up' - this table needs to focus on the participants for whom outcomes are available and are included in the comparison of treatment groups. Tables 1 and 2 which compare characteristics of women who are included and not included are addressing a secondary question which, although interesting and even crucial in this case given the large amount of losses/attrition, does not belong to this section of the results.
- a results table showing the counts and proportions of participants with each binary outcome in each treatment group, along with unadjusted and adjusted effects (odds ratios) with 95% confidence intervals and p-values (and no other information, i.e. the main results) should be presented (e.g. Table 4 in https://www.sciencedirect.com/science/article/pii/S0140673618317823 ignoring the 'risk difference' column.)
- (notwithstanding my comment above about the relevance of the analysis presented in Table 4) the Wald test p-values in the 5th and 7th columns for age group, ethnicity and education level should be replaced with overall (e.g. likelihood ratio) p-values, as these are the appropriate statistic to evaluate whether each variable is associated with screening attendance. The currently presented Wald test p-values only tell you which level of each variable is different from the comparison category, and they change depending on the comparison category, and don't tell you anything about the overall association between each variable and screening attendance.
- The results in Table 5 are inappropriately presented (and this stems from the inappropriateness of the analytical approach - see my suggested analytical approach above). The appropriate way to present this would be to have the mean and standard error (not standard deviation) of each outcome measure by treatment arm, followed by the unadjusted and adjusted mean differences with 95% confidence intervals and p-values, for example Table 2 in https://www.sciencedirect.com/science/article/pii/S0140673618317823. The columns indicating the group means and SEs should indicate the numbers of individuals included in the analysis.
- It is not clear which objective the analysis presented in Table 6 is addressing.
- There is a lot of attrition/losses in this trial and the number of participants eventually included in the analysis is probably smaller than what was required under the original assumptions (see my comment on the sample size calculation). A clear recognition of this should be made in the manuscript, and this is the part where some analysis of how the individuals included versus not compare (i.e. Tables 1 and 2) belong, but only at the end of the results after the analyses addressing the main objectives have been presented in a standard manner.

Overall comments
This is an important study. However, analysis and reporting need to be improved. I would suggest that the authors use the CONSORT checklist to aid their reporting, that they focus the presentation of the analysis and results on the objectives of the trial, to report the results in a manner consistent with what is expected of clinical trial reports, and to clarify their sample size calculation, addressing the issues I have raised.
