# Supplementary material for: The development and evaluation of a mHealth, community education and navigation intervention to improve clinical breast examination uptake in Segamat Malaysia: A randomised controlled trial
Source: PLoS One. 2023 Oct 5;18(10):e0288437. doi: 10.1371/journal.pone.0288437 (PMC10553222; doi:10.1371/journal.pone.0288437)
Supplement: Attachment — Submitted filename: Response to reviewers.docx [file pone.0288437.s007.docx]

Dear PLOS One Editorial Team,

Many thanks for giving us the opportunity to revise our manuscript entitled, ‘The development and evaluation of a mHealth, community education and navigation intervention to improve clinical breast examination uptake in Segamat Malaysia: a randomised control trial’. We would like to thank the Editor and Reviewers for their comments on this manuscript. We have attempted to address all comments as outlined below and highlighted in the marked copy of the manuscript.

If there are any further questions or suggestions for improvement, please do not hesitate to get in touch.

Kind regards,

Dr Desiree Schliemann

(on behalf of the study team)
**Journal Requirements:**

Please review your reference list to ensure that it is complete and correct. If you have cited papers that have been retracted, please include the rationale for doing so in the manuscript text, or remove these references and replace them with relevant current references. Any changes to the reference list should be mentioned in the rebuttal letter that accompanies your revised manuscript. If you need to cite a retracted article, indicate the article’s retracted status in the References list and also include a citation and full reference for the retraction notice.

**Response:** We have revised the reference list and removed any duplicate references. We did not include any retracted articles.

**Additional Editor Comments:**

Pleased clarify the sample size calculation in details.

A few lingering issues:

- the sample size calculation is still not clearly reported; it seems to suggest a total sample size of 932 for both 80% power and 90% power, which isn't right. Also, the authors indicate that the sample size was adjusted for losses (attrition, etc). You need to specify what adjustment was made for losses, e.g. 10%? The statement also needs to be clear about whether this calculation refers to the total number of individuals required, or the number of individuals required per group.

**Response:** We have updated the sample size calculation.

- still on sample size, the statement reporting the number of women randomised (line 261: "IG (n=888 or CG (n=881)" is a result and belongs to the results section, if not already reported there. IT should be removed from here.

**Response:** We moved this from the methods into the results section.

- the results now seem well-reported; to pay attention to bits such as in line 311 and 315 where a character (I believe the 'chi' character) is missing.

**Response:** We have included the ꭓ (Chi-squared) symbol in the Word document but it may have gotten lost when the document was converted into a PDF document. Hopefully the editorial team is able to include the chi-squared symbol from the Word documents in the final publication.
